# Fetal Programming Is Deeply Related to Maternal Selenium Status and Oxidative Balance; Experimental Offspring Health Repercussions

**DOI:** 10.3390/nu13062085

**Published:** 2021-06-18

**Authors:** María Luisa Ojeda, Fátima Nogales, Inés Romero-Herrera, Olimpia Carreras

**Affiliations:** Department of Physiology, Faculty of Pharmacy, Seville University, 41012 Seville, Spain; ojedamuri11@us.es (M.L.O.); inesrh@hotmail.es (I.R.-H.); olimpia@us.es (O.C.)

**Keywords:** selenium, oxidative stress, fetal programming, selenoproteins, fetal alcohol spectrum disorders, intrauterine growth retardation

## Abstract

Nutrients consumed by mothers during pregnancy and lactation can exert permanent effects upon infant developing tissues, which could represent an important risk factor for diseases during adulthood. One of the important nutrients that contributes to regulating the cell cycle and tissue development and functionality is the trace element selenium (Se). Maternal Se requirements increase during gestation and lactation. Se performs its biological action by forming part of 25 selenoproteins, most of which have antioxidant properties, such as glutathione peroxidases (GPxs) and selenoprotein P (SELENOP). These are also related to endocrine regulation, appetite, growth and energy homeostasis. In experimental studies, it has been found that low dietary maternal Se supply leads to an important oxidative disruption in dams and in their progeny. This oxidative stress deeply affects gestational parameters, and leads to intrauterine growth retardation and abnormal development of tissues, which is related to endocrine metabolic imbalance. Childhood pathologies related to oxidative stress during pregnancy and/or lactation, leading to metabolic programing disorders like fetal alcohol spectrum disorders (FASD), have been associated with a low maternal Se status and intrauterine growth retardation. In this context, Se supplementation therapy to alcoholic dams avoids growth retardation, hepatic oxidation and improves gestational and breastfeeding parameters in FASD pups. This review is focused on the important role that Se plays during intrauterine and breastfeeding development, in order to highlight it as a marker and/or a nutritional strategy to avoid diverse fetal programming disorders related to oxidative stress.

## 1. Fetal Programming and Oxidative Stress

The term “fetal programming” (FP) was introduced by epidemiologist David Barker over 20 years ago, who investigated the relationship between low birth weight and increased risk of coronary disease in adult life [1]. FP occurs by the effects of certain hostile factors or stressors on the optimal environment in which the fetus has grown up, especially in the development period of the vital organs. Although the precise mechanisms are not yet known, there is a correlation between intrauterine stress and adverse effects in the offspring for several diseases such as atopic syndromes, increased vulnerability to infections, metabolic dysfunction, cardiovascular disease, and cancer [2]. At present, FP is also called “developmental programming” [3], since this concept also implies that factors affecting fetal growth and development lead to long-term changes in organ structure and/or function later in life [4,5]. In this way, adults can develop several chronic pathologies, including metabolic syndrome, abnormalities in growth and/or alterations in reproduction, immunity or cognitive functions, such as deficits in hippocampal-related behaviors and associated neurogenesis [3,6].

In this sense, the presence of stressors during pregnancy, including nutrients consumed or not by mothers, endocrine maternal alterations such as insulin resistance (IR), young or advanced maternal age, maternal or embryonic genetic problems, environmental stress, or lifestyle choices such as alcoholic consumption, can profoundly compromise fetal postnatal development [3,7]. The influence of intrauterine stress on the offspring depends on the duration and impact of the stressor, as well as on the gestational age at which fetal exposure occurs [8]. One of the main mechanisms related to a FP adverse outcome is oxidative stress (OS). OS is defined as the negative imbalance between pro- and antioxidants elements; it occurs when the amount of reactive oxidative species (ROS) in the cell exceeds its antioxidant defense capacity [9]. The mechanisms that the body has to oppose OS are classified into enzymatic (glutathione peroxidase (GPxs) family, superoxide dismutase (SOD), catalase (CAT)) and non-enzymatic (glutathione (GSH), thioredoxin) endogenous antioxidant systems; and exogenous antioxidants such as vitamins (C, E, folic acid) and metals (zinc, copper, selenium). The main ROS molecules generated by the organisms include the superoxide anion (O_2_^−^), hydroxyl radical (^•^OH) and hydrogen peroxide (H_2_O_2_), as well as other molecules that are generated with reactive molecules, such as NO and peroxynitrite, the so-called reactive species of nitrogen (RNS). These reactive species could lead to lipid, protein and DNA damage, which would compromise cell function [10]. Therefore, OS are responsible for a number of inflammatory, apoptotic, metabolic and growth retardation effects in the offspring [11,12,13]. Specifically, OS affects gestational parameters, leads to intrauterine growth retardation (IUGR) and abnormal development of tissues, which is related to endocrine metabolic imbalance and disorders in pregnancy [14,15,16,17,18,19]. OS effects depend on the ROS generated and the antioxidants present, as well as on the type of cell or organ affected [20]. Since OS is related to multiple diseases during adulthood, including neurodegenerative disease, cardiovascular disease, cancer and renal pathologies, its effects on the FP process should be more deeply studied [21,22,23,24,25].

An appropriate oxidative balance plays a pivotal role during the whole reproductive process: preconception, gestation and lactation periods. It is widely known that in the etiology of male infertility, OS plays an important role, since spermatozoa are highly vulnerable to ROS owing to their restrained levels of antioxidant defense and their single, limited DNA-damage detection and repair mechanisms [14]. Relative to female preconception problems, OS has been implicated in the etiology of the polycystic ovary syndrome, endometriosis and premature ovarian failure, influencing female fertility [26]. Most probably, all the modifications in male and female fertility are due to the fact that male and female germ lines are highly vulnerable to OS [27].

During embryonic development, numerous and complex cellular events occur, such as proliferation, differentiation and apoptosis [7]. Low concentrations of ROS serve as signaling molecules that induce the transcription of several genes (e.g., HIF1A, CREB1, NFKB1), important in oxygen detection, cell differentiation, and cell proliferation [12,28,29]. ROS can also act as second messengers, take part in the immune system acting against pathogens or in the hormesis [20,30,31]. However, a disturbance in the generation of ROS and RNS, an imbalance of the antioxidant defense system, and the disruption of cellular redox homeostasis produce OS and cell damage. OS during the embryonic phase can easily occur, since despite the fact that the development of the embryo arises in a relatively low oxygen environment, it has low antioxidant capacity, being highly susceptible to oxidative injury [32,33].

As the maturation of the placenta progresses, there is a transfer of oxygen to the fetus, which is required to increase the metabolic rate during the phase of rapid fetal growth [34]. It is known that, specifically, basal NADPH oxidase (NOX)-mediated ROS generation is not only increased, but also accompanied by a higher antioxidant capacity in the early pregnancy placenta period as compared to that in the mature placenta period [35]. It has been postulated that the increase in NOX activity may contribute to trophoblast proliferation, spiral artery remodeling and placental growth, and furthermore, it could also stimulate redox sensitive gene expression. However, once more, any complication in oxidative balance could deeply affect placental function by altering an adequate remodeling of spiral arteries by extra villous trophoblasts, affecting the exchange of nutrients and oxygen between mother and fetus. Consequently, this could result in the manifestation of pregnancy complications such as pre-eclampsia and IUGR due to hypoxia [36]. Therefore, during the development of the embryo and fetus, there is a fragile balance between the oxygen levels and the production of ROS [8]. At the end of gestation, a physiologic increase of the antioxidant capacity emerges in order to have a proper preparation for the transition to extra uterine life [37]. Normally, during pregnancy, OS occurs not only due to the fetus development [11], but it also appears due to the maternal physiological state, as a result of a higher metabolic turnover and elevated tissue oxygen requirements [38]; the placenta acts as a key place for the activity of many antioxidants.

Reproduction is the highest energy demand period for mammals, and it finalizes with the breastfeeding period. In this period, both energy intake and expenditure are increased to cope with the elevated energy requirements of the offspring growth and the somatic protection as ROS are usually generated in direct proportion to the metabolic rate. In this reproductive period, OS also plays an important role in mothers and neonates [39]. For instance, it has been proposed that OS is implicated in several health disorders during the periparturient period, such as mastitis and maternal metabolic disorders [40,41,42]. In this context, maternal altered nutrient metabolism can affect neonate growth and the onset of an appropriate lactation process. The higher ROS exposure together with the decreased intake of dietary-antioxidants can lead to a pro-oxidant shift in the redox balance, which compromises offspring immune competence [43,44].

Fortunately, there are nutrients with antioxidant and anti-inflammatory activities, which, included in the maternal diet, could counteract OS. This could be a strategy to inhibit the attack of the ROS produced over proteins, membrane phospholipids, and DNA in mothers and their progeny. One of these important nutrients is the trace element selenium (Se) [16,17].

## 2. Selenium during Preconception, Gestation and Lactation

The trace element Se (Se34 79) is essential for maternal health and fetal development during pregnancy and breastfeeding. This is not only due to its effects on the oxidant/antioxidant balance, but also for its other functions related to the modulation of metabolism, endocrine and energy balance [7,16,19]. Therefore, it is also implicated in protein synthesis, regulation of the cell cycle, remodeling of the tissue structure and its functionality [18,45]. It should be noted that pregnant and lactating women present reduced plasma Se levels, coinciding with the increased demand for this nutrient by the fetus and neonate, which makes its supplementation necessary [46]. According to that, the Se requirements during pregnancy and lactation are increased, reaching 65 μg/day for pregnant women and 75 μg/day for lactating mothers (women: 55 g/day) [47,48]. Recently, it has been shown that during preconception, Se proper levels are also important, since there are studies that relate Se with the growth and maturation of follicles, and that link low Se intake with a longer time to become pregnant [49,50]. However, further research is required on the mechanisms relating Se to periconception health in women, which may provide recommendations to increase maternal dietary Se intake in women planning a pregnancy. Thus, Se deficiency in women has been associated with miscarriages, premature births, infertility, thyroid dysfunction, diabetes and impaired bone metabolism [51,52,53,54].

The beneficial role of Se in health is due to its relationship with selenoproteins, which include 25 proteins with at least one selenocysteine (Se-Cys), a selenium-containing amino acid, and most of them have antioxidant properties such as the GPxs family (GPx1–GPx8) and selenoprotein P (SELENOP), which are also related to endocrine regulation, appetite, growth and energy homeostasis [55]. Selenoproteins also participate in different processes, including the regulation of thyroid hormones (iodothyronine deiodinase family (DIOs: 1, 2 and 3 families), redox regulation processes (thioredoxin reductase family (TXNRDs: 1 and 2), SelW, SelH, SelT, SelV), and the regulation of apoptosis (SeP15) [47].

The main functions of selenoproteins, which are implicated to have a role in fertility, reproduction and development, are described by Qazi et al. [56]. GPx1 plays a pivotal role in female reproductive function due to its antioxidant properties in the follicular microenvironment and in the follicle. GPx2 has antioxidant functions in the embryonic and extra-embryonic tissues. GPx3 is implicated in the implantation in the endometrium, in the maternal-fetal Se transfer mechanism, in common pregnancy and birth, in pre-eclampsia, and in the growth of large healthy follicles. GPx4 is vital for the embryonic development and male fertility. GPx5 has an antioxidant role during sperm maturation. SELENOP is implicated in the maternal-fetal Se transfer mechanism, male fertility, and in sperm development. TXNRD1 and TXNRD2 have important roles in embryogenesis. DIO2 and DIO3 have a high expression and activity in the implantation of pregnant rats, and DIO3 has a role in the utero-placental unit and fetal epithelia. SelS is remarkable to pre-eclampsia and SelV for the testis of rodents.

Depending on the Se dose and form, Se may exert a toxic effect at low and high doses, which is called the U-shaped response; only adequate Se dietary intake provides beneficial effects on health [57]. However, selenoproteins are generally considered beneficial for health, although GPx1 is implicated in insulin resistance (IR) (Zhou et al., 2013) and SELENOP is associated with type 2 diabetes mellitus (T2DM) [58,59].

Therefore, during the reproductive period, when OS plays a pivotal role, Se and the antioxidant selenoproteins, among others, play a crucial role for a correct FP process. Regarding this, in experimental studies, it has been found that an appropriate dietary maternal Se supply is necessary for a proper oxidative balance in dams and in their progeny [7,60]. Two selenoprotein-dependent maternal-fetal Se transfer mechanisms have been well identified during early pregnancy, through the visceral yolk sac and thereafter through the placenta [61]. From early to mid-gestation, the visceral yolk sac takes up both SELENOP (plasma Se transport protein) and GPx3 (main antioxidant in plasma) from the uterine fluid by bulk pinocytosis. In the second half of gestation, the placenta takes up maternal Se, not only by SELENOP from the maternal circulation via apoER2-mediated endocytosis, but also by GPx3 [62]. It is known that Se levels, GPx3 and SELENOP activities are higher in the mother’s serum as compared to the cord serum. These differences cannot be explained by simple diffusion; it is probably that a specific transfer mechanism is implied. It seems that at delivery, GPx3 concentrations in mothers are related to maternal Se status, whereas the GPx3 activity in cord serum depends on gestational age [63]. Studies in mice and rats suggest that there is another pathway involved in the transfer of Se to the fetus independent to the pathway of the extracellular selenoproteins, through methionine transporters, such as the proteins that contain selenomethionine (SelMet) or SelMet itself [64]. This mechanism is not regulated and depends on the amount of SelMet in the mother’s diet; therefore, it will be less effective in conditions of Se deficiency [61]. Placental tissue expresses different selenoproteins such as SELENOP, GPx1, GPx4 and TXNRD, which play a crucial role protecting trophoblast cells from mitochondrial OS [65]. Se supplementation increased mitochondrial content and up-regulated mitochondrial biogenesis mediators in cells [66]. As a result, Se increases endogenous antioxidant expression of GPx and TXNRD1, suggesting that the imbalance of the mitochondrial OS in trophoblasts can be established with Se supplementation [65]. This antioxidant effect is essential since placental development is highly dependent on oxygen status. Furthermore, uncontrolled ROS formation is probably detrimental [51], since OS is related to placental apoptosis/necrosis, leading to the pathogenesis of many disorders during pregnancy [65,67].

It is also interesting to point out that GPx4 knockout mice are non-viable, since embryos died by gestational day E 8.5 [68]. It could appear that GPx4 depletion, in mice, leads to cell death in embryos’ testis, brain, liver, heart and photoreceptor cells by lipid peroxidation and oxidative modifications of the mitochondrial cardiolipin, which in turn facilitates cytochrome-c release and activates the apoptotic signaling cascade [69]. The suppression of phospholipid peroxidation by antioxidant compounds is pivotal to cell survival in normal tissues in mice [69]. Moreover, GPx4 is also related to the activation of the transcription factor essential for inflammatory responses, nuclear factor-kB. Dysfunctional NF-kB expression causes embryonic mortality around gestational day E-15 [70]. Furthermore, it has also been found that inactivation of TXNRD1 leads to early embryonic lethality, displaying severe growth retardation and playing an essential role in most developing tissues except for the heart [71]. The main antioxidant enzyme in the rodent gastrointestinal tract is GPx2, being a first line of defense against gut-derived ROS. However, during organogenesis, it seems to play more roles, since GPx2 is expressed more amply in the extraembryonic tissues including placenta, than in embryos [72]. Se has already been proposed as a possible therapeutic agent for cardiovascular problems and neuron survival in embryos [62,73]. Due to all of these actions, it is easy to understand that Se plays a pivotal role in embryogenesis and in the early stages of life.

Despite the fact that the main action of Se during the embryo stage is related to GPxs antioxidant activity in different tissues, other authors have found that Se plays an important role during fetal development related to growth and metabolic endocrine function [60]. It has been suggested that a correct Se status can prevent the risk of IUGR, tissue underdevelopment and signs of liver oxidation, not only by increasing GPxs activity, but also by its relationship to normal growth in children. In this context, Se improves duodenal function and influences endocrine secretion of different hormones deeply related to early growth, such as thyroid hormones (THs), insulin-like growth factor (IGF) and insulin [18,74,75].

During the lactation period, the maternal Se requirement is even higher than during pregnancy, since in this period energy intake and expenditure are enhanced to cope with the progeny needs, which is intimately related to ROS generation, and antioxidant selenoproteins’ actions are required [39]. During breastfeeding, Se is especially and extremely important for neonates, since during pregnancy Se is transferred through the placental barrier and, even if there is a moderate maternal Se deficient status, the offspring receives a sufficient Se supply. However, in the first weeks of life, milk is the only source of Se for the neonate, but the Se content of milk is rather low. Consequently, pups from dams with insufficient Se supplementation may suffer disorders related to Se deficiency [60]. Moreover, unlike other trace elements, Se concentration in milk depends on maternal status or intake [76]. Therefore, a proper Se supply in maternal milk is necessary for the progeny’s health. Its concentration is especially high in colostrum, decreasing as lactation progresses, and depending on maternal nutritional status. It is known that maternal Se intake has an effect on breast-milk Se concentration; in this context, the mechanism of Se complexation with S-containing amino-acids modulates Se incorporation into milk proteins. Total Se secretion via breast milk depends on these milk proteins and explains its fall as lactation progresses, especially after the protein-rich colostrum. GPx seems to be the main selenoprotein in breast milk. Se prophylaxis raises maternal Se status and increases both breast-milk Se and GPx activity. The mammary gland secretes Se as Se-containing amino acids in the proteins of milk, protecting the infant from excessive maternal Se intake. Hence, infants fed with breast milk show higher Se than formula-fed infants [77]. However, Se milk composition is also affected by others insults such as subclinical mastitis and the increase of cytokines in human breast milk [78]. Maternal Se supplementation during gestation and lactation increased GPx activity and decreased lipid oxidation in colostrum, improving reproductive performance and body weight at the end of lactation in pigs by enhancing antioxidant capacity and fat content in milk [79]. Breastfeeding pups treated with Se through lactation until weaning improved their spatial memory and hippocampal potentiation by modulating OS and apoptosis in neurons [80]. Finally, Se supplements for heat-stressed or actively cooled sows improved piglet preweaning survival, colostrum and milk composition and oxidative capacity, as well as maternal Se-antioxidant status and immunoglobulin transfer by increasing IgM in colostrum and IgA in milk [78].

For all these reasons, Se is recognized as an essential element to human reproduction. In humans it reduces the time to conceive and improves fertility [50], decreases the risk of hypertension and other pregnancy complications in pregnant women [51,81,82,83,84], and balances important inflammatory indicators such as C-reactive protein and the antioxidant defense system [82,83,85]. Furthermore, Se deficiency affects health parameters in children like growth and development, as well as cognitive functions of infants in the first few years of life [86,87,88]. Consequently, it seems that Se during reproductive periods could be an important marker and an appropriate nutritional strategy to avoid diverse FP disorders related to OS. In this context, our research group has designed several experimental models (Se-deficiency, EtOH-exposure, or EtOH-exposure with Se supplementation during preconception, gestation, and lactation) in order to analyze the role of Se in mothers and their progeny.

## 3. Low Se and Fetal Programming

### 3.1. Fertility, Gestational and Breastfeeding Parameters

As mentioned before, during preconception and gestation periods, a maternal Se deficient diet can cause complications both in mothers and fetus. Some of these problems may be pre-eclampsia, glucose intolerance, alterations in the lipidic profile, IUGR, mental and psychomotor delay, premature birth or miscarriage [60,88,89,90,91].

**Figure 1:**
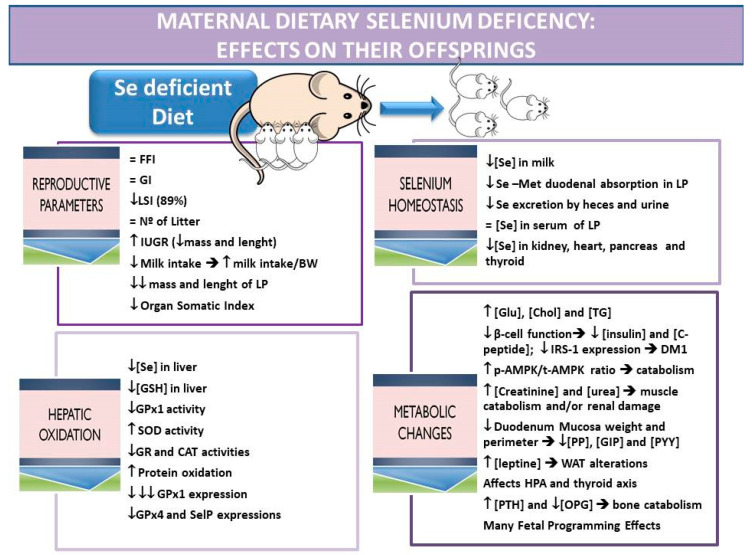
Maternal dietary selenium deficiency: effects on their offspring related to reproductive parameters, selenium homeostasis, hepatic oxidation and metabolic changes. The results are extracted from a Se-deficient dietary experimental model applied to mothers during preconception, gestation and lactation periods carried out by this Research Group. FFI: fertility female index (number of pregnancies/number of mating × 100), GI: gestation index (number of successful births/number of pregnancy rats × 100), LSI: lactation survival index ((number of total offspring—number of died offspring/number of total offspring) × 100), IUGR: intrauterine growth retardation, BW: body weight, LP: lactating pups, GSH: glutathione, GPx: glutathione peroxidase, SOD: superoxide dismutase, CAT: catalase, GR: glutathione reductase, SelP: selenoprotein P, Chol: cholesterol, TG: triglycerides, Gluc: glucose, IRS-1: insulin receptor sustrate-1, DM1: type 1 diabetes mellitus, HPA: hypothalamic–pituitary–adrenal axis, PP: pancreatic polypeptide, GIP: gastric inhibitory polypeptide, PYY: peptide YY, PTH: parathyroid hormone, OPG: osteoprotegerin, AMPK: AMP-activated protein kinase, WAT: white adipose tissue.

Maternal dietary Se interventions lead to different Se concentration values in colostrum, milk and offspring’s blood depending on the source of Se used (organic or inorganic), the Se dose used, and the duration of treatment [92]. Therefore, throughout this review, the results obtain by the Se-deficient model (SD) carried out in our laboratory will be primary analyzed. This experimental model uses a control standard diet, which contained 0.1 ppm of Se as sodium selenite, and a Se-deficient diet, which contained 0.01 ppm of Se from a sodium selenite source. These diets were given to dams during preconception, pregnancy and lactation periods [60,74]. SD-exposure did not alter the reproduction function of the parents as reflected in the indices of female fertility and gestation and the number of offspring per litter (Figure 1) [60]. This may be because the SD diet received during preconception (3 weeks) takes a longer time to repercuss in Se gonadal deposits. Moreover, the dams exposed to SD diet ingested a large amount of food prior to and during pregnancy, probably to increase their Se deposits, transferring more of the Se to their offspring [17,60,74]. However, despite the efforts to provide an optimal amount of Se (enough for fetus survival), SD mothers gave birth to an extremely low birth weight and cranial-caudal length offspring. These results confirm that Se is an essential trace element to avoid IUGR, probably due to its antioxidant effects on placenta, since SD-dams consumed a higher amount of food, where Se was the only nutrient not acquired in adequate quantity during the reproductive period. Hofstee et al. [18] also demonstrated that Se deficiency in mice induces growth restriction, with significant diminution of fetal organ weights, such as the heart and kidney, which may predispose to cardiovascular and renal alterations in later life. These authors affirmed that the Se deficiency can dysregulate the placental nutrient transporter expression, decreasing fetal glucose concentration, which contributes to the so-called growth restriction. In addition, Se influences fetal development by modulating TH homeostasis through the activity of DIOs, specifically DIO2, which transforms tetraiodothyronine (T4) into the bioactive thyroid hormone triiodothyronine (T3) [18,60,75].

With regard to the breastfeeding parameters, maternal SD exposure during the entire experimental period reduced viability and survival indices (<89%) at the end of lactation, and decreased pup development (Figure 1). SD nursing mothers ingested a high amount of food with a very low Se content, having higher serum Se levels than control dams by sacrificing their own Se tissue reserves at the end of lactation [93]. However, the Se content in milk did not reflect the increase in serum Se values, and lactating pups, which consumed less milk than control ones, received lower amounts of Se by milk [17,60]. Since Se concentration in breast milk decreases as lactation progresses, and SD-pups had not received a proper Se supply during gestation, lactating survival indices were affected, leading in some cases to pups’ death. Moreover, at the end of weaning, SD-offspring showed a great reduction in length and mass (higher than at birth), together with a reduction in liver, pancreas, and thyroid mass. For all these reasons, a Se-compromised status particularly affects the lactation period development. As explained before, it is well established that a proper Se amount is necessary during the reproductive period to guarantee an adequate transference of this mineral, via placenta or mammary glands, to pups [18,88,91,94]. However, during the lactation period, another tissue related to Se absorption is compromised, the gastrointestinal tract (GIT), since this tissue has to adapt itself to the new functions it would lead after birth. In this context, SD-weaning pups present intestinal development restriction, decreasing mucosa weight and intestinal perimeter [74], and present severe alterations in GIT-peptides related to appetite and energy homeostasis [93]. These effects could be in part because Se is crucial in the growth and maturation of the duodenum, since it is part of the GPx2, which protects intestinal cells from OS and modulates enteroendocrine cells [95]. Recently, He et al. [96] found that a Se deficient diet in chickens decreases the villus length, crypt depth, mucosal thickness and GPx activity through the GIT, increasing OS and the risk of contracting diseases. These alterations severely affected the structure and function of the intestine, decreasing the chickens’ digestive absorption and resulting in weight loss. Moreover, not only are food intake and absorption compromised in SD-weaning pups, but they also present a catabolic general profile that limits tissues growth and development [93]. These results point to Se as a pivotal mineral in the regulation of growth, food intake, energy and metabolism during breastfeeding. These SD offspring presented low serum thyrotropin (TSH) levels; however, they had normal serum T3 and T4 levels. Thus, these pups had an altered thyroid axis, compromising cell proliferation and metabolism, and therefore growth [18].

### 3.2. Selenium Homeostasis

Newborns, especially those born at an adequate gestation time, presented more Se reserves than pre-term offspring since the buildup of this mineral occurs in utero at the end of gestation. Generally, these reserves are low and the newborn must acquire more Se via maternal milk [91,97]. In this context, since SD-pups intake a lower amount of Se by milk, present a down-regulation of SelMet duodenal absorption [74], and had not received proper Se supply during gestation, they presented extremely low deposits of Se in liver, kidney, heart, thyroid, and pancreas, affecting the homeostasis of the selenoproteins in those tissues and their function [17,60,74,93].

These SD-offspring tried to maintain Se homeostasis by decreasing the excretion of this element in feces and urine. Thus, despite the fact that they ingested less milk with a lower Se content after 30 min of suckling, these SD pups presented normal serum Se levels at the end of lactation [74]. This is probably due to an attempt to maintain high levels of Se in the plasma in order to quickly derive it to the tissues with more Se demand to oppose OS [98].

### 3.3. Hepatic Oxidation

In the SD experimental model used, both dams and their offspring presented an important hepatic oxidative disruption [60]. The liver is the main organ related to the general body metabolism and the main secretor of IGF-1, a mitogenic hormone involved in many processes such as growth, metabolism, angiogenesis and differentiation, which plays a crucial role in fetal development. Therefore, the hepatic OS provoked by low Se supply is responsible in part for the altered reproductive parameters, the IUGR and the underdevelopment status observed in pups, which are also joined to the endocrine energy imbalance during early programming.

The SD-model during preconception, gestation and lactation produced an important Se depletion in the liver of pups, and thus a decrease of activity of the GPx selenoproteins, which form part of the main antioxidant defense system against OS and H_2_O_2_ generation in this tissue (Figure 1) [60]. Moreover, the hepatic GPx1 selenoprotein expression in SD-pups is deeply decreased, demonstrating that it is closely related to GPx activity and hepatic Se levels. Sherlock et al. [99] recently studied specific neonatal selenoprotein expression in response to limited maternal Se supply. They found that low Se-maternal supply during preconception and gestation reduced plasma and hepatic GPx activity, as well as the expression of GPx1 in the liver. However, these authors observed that low Se-maternal diet affected neither other selenoproteins in this tissue nor the GPx activity in other organs.

The SD-model, during the whole reproductive period, also reduced the hepatic expression of selenoproteins GPx4 and SelP. GPx4 is a selenoprotein with essential functions for life, especially during embryonic, fetal and breastfeeding periods, since it reduces hydroperoxides in lipoproteins and complex lipids and protects mitochondrial membranes from oxidative damage [100]. However, the decrease found in GPx4 expression was lower than the one observed in GPx1 expression. This fact was possible because GPx1 is the selenoprotein most dependent on dietary Se in adult animals [101]. The same should occur in these SD-pups. In this context, since GPx4 protects newly synthesized phospholipids from oxidation, and these phospholipids are necessary for a correct cell-membrane structure, hepatic Se deposits are transferred to maintain GPx4 expression. Hepatic SELENOP also decreased during SD-exposure in a minor proportion compared to GPx1. Since this selenoprotein plays a vital role in Se transport to blood and the rest of the tissues, in serum, Se levels were not depleted in SD-pups at the end of weaning [102,103,104,105]. Moreover, the hepatic SELENOP has been considered a hepatokine, which, modulating the energetic sensor AMP-activated protein kinase (AMPK) in liver, interferes with the insulin signaling cascade, decreasing the activation of the insulin receptor substrate (IRS-1) [106]. As it will be described in the next point, AMPK hepatic activation is also affected in SD-pups at the end of lactation.

According to the lower expression of GPxs found in liver, SD nursing pups presented oxidation in hepatic proteins as well as a lower GR and CAT activities; however, they had a higher SOD activity. Numerous studies have observed a concomitant increase in SOD activity in low Se exposed-adults [107,108] and pups born to dams exposed to a longer duration of Se-deficiency [99]. This increase in SOD activity was related to the hepatic oxidation found in SD pups due to a higher produced amount of H_2_O_2_. This ROS could not be removed from the liver since GPx and CAT activities were low, which increased the Fenton reaction, oxidizing hepatic proteins. Nogales et al. [60] suggested that there was a clear relationship between SD treatment, GPx activity and protein oxidation in liver, which also correlates with a low liver development.

### 3.4. Metabolic Changes

Se deficient supply during the reproduction period has been related to metabolic disorders, since Se deposits and selenoprotein expression are decreased in different tissues (including endocrine glands) of weaning SD-pups [17]. Thus, the SD-model deeply reduced Se levels in the pancreas of lactating pups. This trace element is necessary for correct development and functioning of the pancreas, and is responsible for the low β-cell function and almost null insulin secretion observed in SD pups at the end of lactation [93]. In addition, Se restriction during the gestation and nursing periods provoked a decrease in the levels of the insulin precursor C-peptide, as well as in the levels of the insulin mimetics glucagon and incretins (GLP-1 and GIP) (Figure 1). These data agree with the low IRS-1 expression found in their livers (which is downstream in the insulin signaling pathway), and with the high glucose (glu) plasma levels found in SD-pups. These data suggest that the metabolic profile established by SD-exposure in pups is closely related to a type 1 diabetes mellitus (T1DM) [17,93]. Several studies have found pancreatic atrophy, hypoinsulinemia and glucose intolerance after Se restriction together with a decreased GPx1 and SELENOP expression, as well as OS [109,110,111,112].

Moreover, in the liver, the energy status sensor AMPK is increased in SD-pups. This sensor, which is inversely proportional to SELENOP expression, stimulates catabolic pathways in SD-pups, favoring the high levels of triglycerides (TG) and cholesterol (chol) that these rats present [17]. These results were also consistent with the high serum creatinine and urea concentration found in these pups, which indicated renal problems and/or the occurrence of catabolism in the muscle.

Global energy metabolism regulation is carried out in the hypothalamus. The hypothalamic arcuate nucleus (ARC) has been defined as a primary integrator of peripheral metabolic and hormonal signals that are divided into long-term and short-term endocrine energy balance signals. The ARC is responsible for relaying energy status information to other brain regions in order to drive changes in appetite, consumption or ingestion behaviors [113]. Short-term signals with anorexigenic effects include gastrointestinal peptides GLP-1, GIP, peptide YY (PYY), and pancreatic polypeptide (PP). These neuroendocrine signals decreased in SD pups according to the underdevelopment of the duodenal mucosa found, and were responsible not only for the high TGs and chol levels observed, but also for the weight loss and the relatively higher milk intake shown at the end of lactation. These results suggested that these short-term signals are down-regulated. On the other hand, insulin and leptin are long-term signals of energy stored and decreased appetite. As previously mentioned, insulin levels are clearly decreased in SD-pups; on the contrary, leptin, which is secreted by the white adipose tissue, is greatly increased in SD weaning rats [93]. Nonetheless, these pups showed leptin resistance, since they had an increase of appetite with a relatively higher milk intake and a lower development of white adipose tissue, muscle, and bone tissues. It could be suggested that SD-diet provoked leptin resistance by diminishing the levels of selenoproteins, such as GPx4 in the ARC, leading to OS. It is known that OS and endoplasmic reticulum stress in the ARC disrupt leptin sensitivity in this nucleus [114]. Since Se deposits in SD-pups were depleted in almost all tissues studied [17,60,74], this depletion could also occur in the ARC of SD-pups, and OS could appear in this nucleus, decreasing its responsiveness to leptin [113].

Relative to bone turnover, previous results agree with the higher parathyroid hormone (PTH) and low osteopontin (OPN) levels found in the serum of SD pups. Since both high PTH and low OPN parameters are related to bone destruction and resorption, it indicates that bone loss is taking place and that leptin resistance is presented in these pups [93]. These results are in consonance with the extreme bone growth retardation that SD-weaning pups present, linking OS, Se status, endocrine function and length.

Low dietary maternal Se supply also affects the hypothalamic-pituitary-adrenal (HPA) and thyroid neuroendocrine axes (Figure 1). In this way, SD pups showed high serum adrenocorticotropic hormone (ACTH), whereas TSH levels were low. These data revealed dysfunctional long-term endocrine signals, since leptin presents an inverse relationship with serum cortisol and ACTH, and a direct one with TSH levels [115,116]. Although TSH levels were lower in SD-pups, T3 and T4 levels in serum were not affected in these offspring at the end of lactation, suggesting that the TSH hormone did not perform its function properly on the thyroid gland. Once more, these data confirmed that central hypothalamic leptin signals were disrupted; therefore, the long-term endocrine signal for energy balance regulation was profoundly altered after SD-exposure. This leptin resistance process together with the almost null insulin secretion found, the higher hepatic OS and AMPK activation, and the general catabolic process described, point to Se as an indispensable element during gestation and lactation, with important repercussions in the fetal programing outcomes.

## 4. Oxidative Stress-Programming Pathologies and Se

According to this, infant pathologies related to OS exposure during gestation and/or lactation that lead to metabolic programing disorders like metabolic syndrome (MS) or fetal alcohol syndrome disorders (FASD), have been reported to be associated with a low Se maternal status and IUGR [117,118,119,120].

In this context, ethanol (EtOH) is a potent pro-oxidant drug with teratogenic effects, since during its oxidative metabolism, which takes place mainly in the liver, a great amount of ROS in the cytoplasm and in organelles (especially mitochondria) is generated, compromising GSH antioxidant levels and antioxidant enzyme activities [55]. These effects generate OS and therefore lipid, protein and DNA damage, as well as mitochondrial dysfunction, all of which are related to disturbances in embryogenesis [121]; in addition, this OS also causes growth retardation and neurotoxicity in the progeny, increasing the incidence of FASD [122]. FASD includes a broad spectrum of disabilities that can result after ethanol exposure during gestation including physical, cognitive, behavioral, emotional and social difficulties [123]. However, according to the fetal programing theory, there is an increasing interest in the effects of maternal EtOH consumption on other organs and systems, and in the increased potential risk for developing chronic conditions such as cardiovascular disease (CVD) or diabetes (DM), in later life. This concept transforms FASD from a neural disorder to a “whole body disorder” [124].

EtOH is metabolized mainly by an oxidative pathway through three enzymes, alcohol dehydrogenase (ADH), catalase, and P450 (CYP2E1), all of which produce acetaldehyde (a toxic compound). Acetaldehyde is rapidly removed by aldehyde dehydrogenase (ALDH), which in turn generates acetate, which has roles both in energy metabolism and in mitochondrial OS generation. Another important enzyme that generates a great amount of ROS directly during EtOH metabolism is CYP2E1 [125]. The liver is the main metabolic organ, being rich in these enzymes; therefore, it plays a pivotal role in EtOH metabolism. However, after alcohol exposure, maternal EtOH hepatic metabolism is not efficient enough, and this drug arrives directly from maternal blood to the fetus in great amounts, remaining longer in both the placenta and the intra-uterine space. Moreover, during gestation, EtOH is metabolized in the placenta and in the liver of the fetus mainly by CYP2E1, generating great amounts of ROS; however, this activity is low compared to adults [126]. In the first trimester, alcohol is metabolized in the placenta [127]; later, at 16 weeks of gestation, EtOH is also metabolized in the liver of the fetus by CYP2E1, and thereafter (26 weeks), also by ADH [128]. Lee et al. [129] used an inhibitor of CYP2E1 resulting in a decrease in growth retardation effects, hyperinsulinemia, liver steatosis and oxidation, mitochondrial dysfunction and macrophage infiltration. Consequently, they demonstrated the importance of the activity of CYP2E1 in the damage provoked by EtOH during these periods.

Moreover, EtOH diffuses through the placenta and distributes rapidly into the fetal compartment [130], where it also has a slower elimination rate (3 to 4% of maternal rate) [131]. Alcohol, therefore, has a particular effect on the fetus due to amniotic accumulation, lower activities of metabolic enzymes and reduced elimination rate. Consequently, prenatal ethanol exposure provokes an increase in OS in almost all the developing organs, affecting their functions [132,133]. Furthermore, the oxidation of the placenta plays a central role in the pathogenesis of many disorders during pregnancy, since it is also related to placental apoptosis/necrosis and lower nutrient arrival to the fetus [67,134]. During the whole reproductive process, maternal EtOH consumption is dangerous; even if mothers drink EtOH during lactation, the ingested EtOH is secreted in the milk, exposing the developing offspring to the pro-oxidant toxic effect of this drug [135].

Therefore, EtOH acts as an acute teratogen not only by generating ROS and decreasing endogenous antioxidant levels, but also by increasing mitochondrial damage; by disrupting neuronal cell-cell adhesion; and by leading to placental vasoconstriction and to the inhibition of cofactors required for fetal growth and development [128]. Like dietary and environmental factors, it also has epigenetic effects mainly via DNA methylation, which alters gene expression; this is a key factor that links prenatal perturbations to a long term programmed disease [136,137,138]. Moreover, maternal EtOH consumption during preconception, gestation and/or lactation also leads to malnutrition in dams, and therefore to undernutrition in their offspring; this results in long lasting effects, which are not a direct effect of EtOH damage [128]. Furthermore, EtOH not only decreases solid intake and nutrient absorption, but it is also a caloric source that provides “empty kcals’’; these nutritional implications are relevant to metabolic programming [139]. This fact is especially interesting if the nutrients affected are those called “exogenous antioxidants”, since some of them are cofactors required to correct the function of the endogenous antioxidant enzymes (SOD, CAT, GR, GPx), such as Cu, Zn, Mn, Fe or Se [140]. Therefore, EtOH not only generates ROS, but it also affects antioxidant enzyme activities, increasing general body oxidation in the progeny.

### 4.1. EtOH: Fertility, Gestational and Breastfeeding Parameters

**Figure 2:**
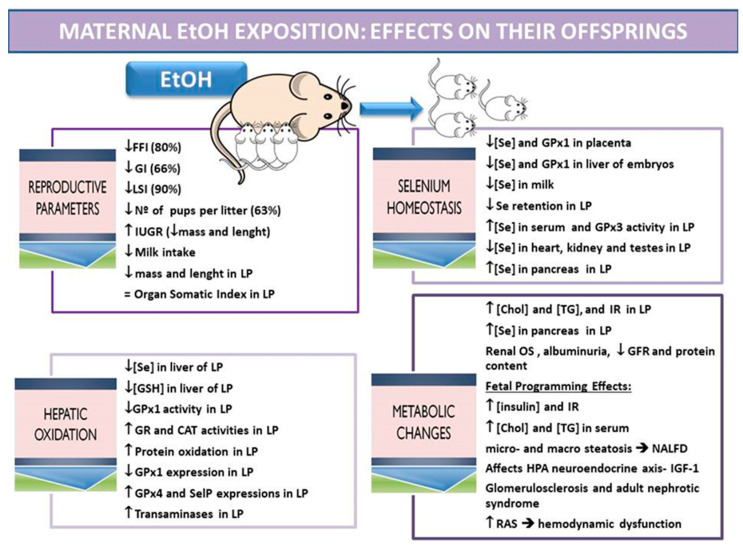
Maternal EtOH exposure: effects on their offspring related to reproductive parameters, selenium homeostasis, hepatic oxidation and metabolic changes. All the results are extracted from a chronic EtOH exposure model applied to mothers during preconception, gestation and lactation periods carried out by this Research Group, except the data included in the metabolic changes section: Fetal Programming Effects. FFI: fertility female index (number of pregnancies/number of mating × 100), GI: gestation index (number of successful births/number of pregnancy rats × 100), LSI: lactation survival index ((number of total offspring—number of died offspring/number of total offspring) × 100), IUGR: intrauterine growth retardation, LP: lactating pups, GSH: glutathione, GPx: glutathione peroxidase, CAT: catalase, GR: glutathione reductase, SelP: selenoprotein P, Chol: cholesterol, TG: triglycerides, OS: oxidative stress, GFR: glomerular filtration rate, IR: insulin resistance, NALFD: non-alcoholic fatty liver disease, HPA: hypothalamic–pituitary–adrenal axis, IGF-1: insulin-like growth factor, RAS: renin-angiotensin system.

In this context, chronic ethanol exposure during preconception and gestation induces an imbalance in the rats reproductive state, decreases the capacity of conception (20%) and of gestation (34%), decreases the number of litters, leads to a reduction in placental development, increasing its oxidation, decreases embryo size, and also increases offspring hepatic oxidation [7]. The IUGR is itself considered a risk for development of future metabolic disorders [141]. All of these effects can become worse for the progeny if the mothers continue drinking ethanol during the breastfeeding period [142]. Despite the fact that there are less studies analyzing the effects of EtOH consumption during breastfeeding and the consequent damage caused to children, it has been found in rats that the pups exposed to EtOH only during weaning presented a lower body weight at the end of breastfeeding than those who were exposed to EtOH only during gestation. However, organ somatic indexes were not altered in both cases [143]. In this context, the lactating survival index after EtOH exposure during breastfeeding was also decreased [7]; these pups have a lower body weight and consume less milk probably because EtOH reduces the suckling process [144] and inhibits prolactin secretion [145]. As it will be seen in the next point, EtOH exposure disrupts Se homeostasis. The lower Se bioavailability found affects its antioxidant and endocrine function, collaborating the IUGR and the growth retardation found in suckling EtOH-exposed pups. Therefore, EtOH exposure during the lactation period needs to be re-evaluated (Figure 2).

### 4.2. EtOH: Selenium Balance

Clinical and research studies have demonstrated that acute or chronic alcohol consumption alters Se bioavailability and its antioxidant function by affecting selenoprotein tissue expressions [146,147]. Nonetheless, few studies analyze Se homeostasis during pregnancy after EtOH exposure [148] in the mother or fetus; however, an impairment of the selenoprotein GPx in FASD during the embryonic period in different tissues is well described [149,150,151,152,153]. Ojeda et al. [154], in an “in vivo” experiment using Wistar rats exposed to chronic EtOH in tap water (20% *v*/*v*) during preconception, gestation and lactation periods (10 weeks), found a decrease in the Se intake and retention in mothers, which lead to changes in their Se tissue distribution, without affecting their serum Se levels. Among others, they presented lower Se deposits in mammary glands and placenta [7], and increased Se levels in liver, probably as a mechanism to increase antioxidant GPx activity, since this tissue is mainly damaged by the pro-oxidative action of EtOH [155]. The lower levels of Se found in mammary glands and placenta were related to a lower milk Se concentration [156] and to lower Se levels and GPx1 expression in the liver of embryos and lactating pups [7].

Additionally, to worsen the relationship among EtOH exposure and Se homeostasis, EtOH-exposed weaning pups intake less milk and have lower intestinal perimeters, presenting a down-regulation in the SelMet transport system [157,158]. This lead to a greater Se homeostasis disruption, more than in dams [159], which mainly compromised Se deposits in liver, kidney and heart. However, these ethanol-exposed pups had higher serum Se levels than control ones and higher GPx3 serum activity [160], as Payne and Southern [98] defend, probably as a compensatory mechanism to maintain plasma GPx activity during periods of low Se intake or oxidative damage.

### 4.3. EtOH: Hepatic Oxidative Balance

Since liver is the main metabolic tissue and it is specially affected by EtOH exposure, the levels of the antioxidant Se should be analyzed. Se was depleted in weaning pups, leading to a lower GPx activity and to a disruption in the antioxidant enzyme balance (GR and CAT activities were increased), increasing protein oxidation and hepatic function, since transaminases were increased [117]. Due to the fact that GPxs selenoproteins have different biological implications, Jotty et al. [156] studied the expression of the three main hepatic selenoproteins (Gpx1, Gpx4 and SELENOP) in the liver of ethanol-exposed pups at the end of lactation. In this context, GPx1 was decreased according to the hepatic Se depletion found, since this cytoplasmic antioxidant protein ranks low in the selenoproteins hierarchy, being dependent on Se status [100]. However, GPx4 expression was up-regulated; it is known that this protein, which prevents cellular phospholipid oxidation and subsequently contributes to correct mitochondria function, plays an important role in embryogenesis, midgestational and breastfeeding periods [69,95,161,162]; for these reasons, its expression is maintained. It is also important to remember that in lactating pups, hepatic phospholipid levels are significantly higher than in adults; in fact, lipid peroxidation does not take place. Like GPx4, SELENOP expression is also increased. This correlates to the higher Se serum levels found in EtOH-exposed lactating pups, since the main function of SELENOP is to deliver Se to the blood in order to distribute it widely to other tissues [163].

### 4.4. EtOH: Metabolic Disorders

Fetal growth restrictions are related to metabolic changes in the long term, such as IR or non-alcoholic fatty liver disease (NAFLD). In this context, prenatal alcohol exposure is known to lead to IR, delaying the hypoglycemic response to insulin but maintaining normal adiponectin levels [164]. Prenatal alcohol exposure also induces dyslipidemia and hyperglycemia in the fetus and the adult offspring, increasing the susceptibility to NAFLD. This is probably due to the intrauterine programming of liver glucose and lipid metabolic function and to the postnatal adaptive catch-up growth triggered by the intrauterine programming of IGF-1 [165]. In a model of EtOH exposure during preconception, gestation and lactation periods, pups presented with higher serum chol and TG levels and renal problems related to OS, such as low protein content, small renal volume, low glomerular filtration rate (GFR) and albuminuria, which can also be related to a future diabetes process [166,167]. These results (higher albuminuria and smaller renal volume) have also been confirmed in children whose mothers drank routinely during pregnancy [168]. Moreover, despite the low Se-status that EtOH-exposed pups present at the end of lactation, Se deposits in the pancreas are increased since Se is necessary for proper insulin secretion, indicating that the insulin-secretion process is altered [159]. Different studies have found in rodents that these effects are maintained later in life, leading to an altered glucose metabolism with high gluconeogenesis, an altered lipid profile, impairments in insulin signaling (since insulin levels are increased) and GLUT4 expression, an increase in resistin serum levels without affecting leptin levels, an imbalance in the plasma lipid profile, which induces liver steatosis, and to an enhanced susceptibility to IR due to the hypothalamic generation of OS, which affects HPA axis-associated neuroendocrine metabolism [169,170,171,172,173]. Similar results have been found in humans [174,175]. Some of these effects have also been confirmed when pups were exposed to EtOH only during the lactation period [170]. Moreover, since the kidney is the second organ implicated in EtOH metabolism and it is highly related to CVD generated by the IR-process [176], different authors have described renal programming problems after maternal EtOH exposure during gestation in their offspring, such as nephron malformations, which could compromise renal hemodynamic functions and cardiovascular function, since a low nephron endowment is considered a risk factor for the development of kidney disease in adulthood [177,178,179,180,181]. In this context, Akison et al. [180] showed that maternal EtOH exposure during pregnancy results in impaired kidney development and leads to a permanent nephron deficit, which has an impact on the adult kidney function. He et al. [182] also found that prenatal EtOH exposure leads to fetal kidney dysplasia and adult glomerulosclerosis in the offspring rats, and that the intrauterine programming alteration of IGF-1 might be involved in fetal-originated glomerulosclerosis. Zhu et al. [183] also suggested that prenatal EtOH exposure induces fetal kidney developmental retardation and adult nephrotic syndrome, by a direct regulation of EtOH through the renal renin-angiotensin system (RAS), contributing to renal hemodynamic dysfunction and CVD generation.

In order to reinforce the hypothesis that EtOH-exposure during preconception, gestation and lactation periods affects the oxidative profile and Se homeostasis in the progeny, which is in part related to their metabolic future health, in our laboratory, dietary Se antioxidant supplementation (0.5 ppm of sodium selenite) was given to EtOH–exposed Wistar dams. With this model, we pursued different objectives: to analyze the reproductive parameters and the intestinal SelMet absorption process in weaning pups [158], to evaluate Se retention and body distribution in dams and their offspring [155,159,184], to improve fetal and lactating pups hepatic oxidative balance, in part related to gestational and breastfeeding parameters [7,154,160,166], and to study selenoprotein liver expression and their possible biological role in weaning pups [156]. The narrow window between therapeutic and toxic doses of Se, as well as the dependence of its effect on the applied form, dose and method of treatment, makes the choice of an effective supplement a very complex issue. In this case, an inorganic form of sodium selenite has been used, since it is the most common source present in the diet [185].

### 4.5. EtOH + Se: Fertility, Gestational and Breastfeeding Parameters

**Figure 3:**
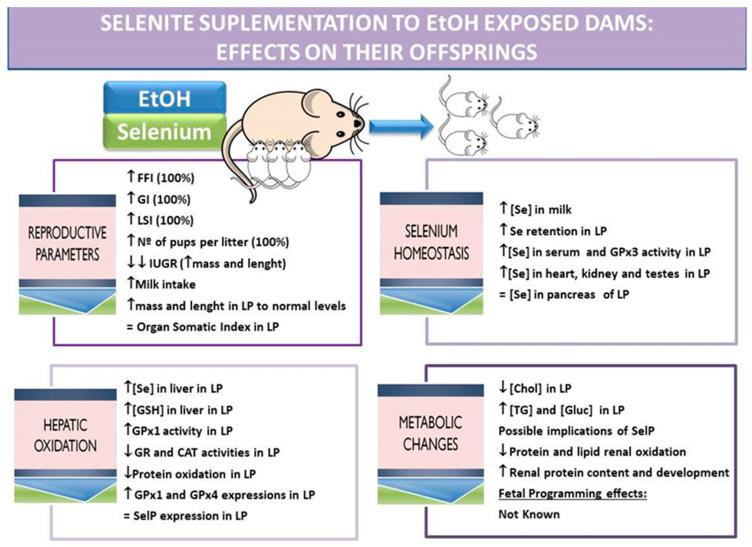
Dietary selenite supplementation to EtOH-exposed dams: effects on their offspring related to reproductive parameters, selenium homeostasis, hepatic oxidation and metabolic changes. The results are extracted from a chronic EtOH exposure model supplemented with Se in the diet applied to mothers during pre-conception, gestation and lactation periods carried out by this Research Group. FFI: fertility female index (number of pregnancies/number of mating × 100), GI: gestation index (number of successful births/number of pregnancy rats × 100), LSI: lactation survival index ((number of total offspring − number of died offspring/number of total offspring) × 100), IUGR: intrauterine growth retardation, LP: lactating pups, GSH: glutathione, GPx: glutathione peroxidase, CAT: catalase, GR: glutathione reductase, SelP: selenoprotein P, Chol: cholesterol, TG: triglycerides, Gluc: glucose.

The Se supplementation used in dams restores, at least in part, the weaning pups Se-Met duodenal absorption; it enhances the affinity of the transporters for Se-Met, and decreases the damage caused by EtOH to the duodenal mucosa, increasing the duodenum perimeter and mucosal development [158]. These improvements are probably due to the increase in duodenal GPx2 expression and activity. This selenoprotein is highly expressed in the gastrointestinal epithelium, and defends it against oxidation and inflammation [186], improving duodenal development and contributing to nutrient absorption. Therefore, the final body weight and length at the end of lactation is clearly increased without affecting body mass index (BMI). These effects on the duodenum are not measured during gestation periods, but dietary maternal Se supplementation during preconception, gestation and lactation periods all improved the fertility, gestational and lactating indexes affected by EtOH consumption, including IUGR (Figure 3). Therefore, in these offspring, Se supplementation plays a pivotal role related to growth, not only by improving duodenal function, but also by leading to a better antioxidant balance (explained in the next point) and by probably improving the endocrine secretion of hormones related to early growth (THs, IGF-1, insulin and leptin).

### 4.6. EtOH + Se: Selenium Balance

With respect to Se homeostasis, the dietary Se supplementation applied to EtOH-exposed dams enhanced the apparent Se absorption rate and retention in dams and in their offspring; in this context, Se intake was increased and its fecal and urine excretion decreased. Pups intake a great amount of milk, and the milk has a greater amount of Se, among others, because the Se-therapy used increased maternal Se deposits in their mammary glands [155]. Therefore, Se body distribution in pups was restored in all the tissues analyzed, even increasing serum GPx3 activity and the plasma antioxidant capacity [117].

### 4.7. EtOH + Se: Hepatic Oxidative Balance

Maternal Se supplementation protects offspring hepatic tissue against the oxidative damage provoked by ethanol exposure during preconception, gestation and lactation by increasing Se deposits, GPx activity and modifying the activities of scavenging enzymes CAT and GR [117]. This therapy mainly acts by increasing GPx1 expression and activity, which was down-regulated in EtOH-exposed pups, improving cytoplasmic oxidative balance in hepatocytes [156]. However, this therapy not only acts like that, it also increases, even more, GPx4 liver expression, which in turns avoids mitochondria oxidation and phospholipid oxidation. This selenoprotein, by defending mitochondria from OS, has anti-apoptotic and anti-inflammatory properties, and is essential in embryogenesis [68]. SELENOP expression is also increased in Se-supplemented EtOH-exposed pups, but this increase was not greater than those found in EtOH-exposed pups. Therefore, EtOH exposure “per se”, regardless of liver Se deposits, up-regulates the expression of this protein, probably to send a higher amount of Se to the blood, in order to have enough Se available to supply it to the tissue that may need it. However, SELENOP has been recently recognized as a hepatokine, which impairs insulin signal transduction and dysregulates glucose metabolism. This leads to glucose intolerance in vivo mainly by affecting AMPK activation [106]. In this way, more metabolic research is needed to know if this protein plays this role in FASD.

### 4.8. EtOH + Se: Metabolic Disorders

Dietary maternal selenite supplementation to EtOH-exposed dams during pre-conception, gestation and lactation decreases the hypercholesterinemia found in EtOH-exposed pups, but leads to hyperglycemia and hypertriglyceridemia [167]. In this offspring, renal parameters were also analyzed, and it was found that Se supplementation improves renal development and protein content. Furthermore, it modifies antioxidant renal enzymes’ activity, including GPx, decreasing lipid and protein nephron oxidation. However, it does not modify the low GFR that EtOH-exposed pups presented [166]. Apart from these results and the studies presented above, little is known about the metabolic implications of Se supplementation during the embryo or post-natal stages under EtOH exposure. Moreover, EtOH leads to epigenetic modification mainly by promoting DNA methylation, which could be implicated in a long-term programmed disease. Se is one of the micronutrients that contribute to modulating one-carbon metabolism; therefore, its supplementation could play a part in balancing DNA methylation. Se is related to one-carbon metabolism because it affects different pathways in the liver, such as the methylation pathway through the modulation of DNA methyltransferases (DNMTs) [187]. Another pathway affected by Se is the transsulfuration, since it uses GSH as a cofactor of the GPxs selenoproteins, even in the offspring [55]. However, there is insufficient knowledge about Se supplementation to EtOH-exposed dams during different reproductive stages and metabolic programming changes. In this context, Kalishwaralal et al. [188] found in the heart of zebrafish embryos exposed to EtOH and supplemented with sodium selenite, that this therapy reduces cardiac oxidative damage through scavenging ROS, preventing pericardial edema and reducing heart apoptosis and cell death. These effects are relevant to avoid future cardiovascular diseases, which are usually related to metabolic disorders.

In summary, dietary selenite supplementation to dams mitigated the adverse effects of alcohol during preconception, gestation and lactation periods. It improved reproduction parameters and pup development [117,158,184] by enhancing duodenum development and increasing selenoprotein hepatic expression, decreasing hepatic OS in hepatocytes and improving liver function, and probably metabolic balance. This improvement in liver function is especially important for a correct EtOH metabolism process [156]. This confirms that Se maternal status is crucial for correct fetal development, and that the selenite therapy is a cheap exogenous supplementation with a real antioxidant capacity and biological function. This micronutrient becomes especially important after ethanol consumption and during gestation and breastfeeding, the early stages of pups’ lives, and probably for a correct fetal programming process.
